# Aryl hydrocarbon receptor-regulated long non-coding RNAs: implications for glycolipid metabolism and prognosis in hepatocellular carcinoma

**DOI:** 10.3389/fonc.2025.1537481

**Published:** 2025-04-03

**Authors:** Xiaoli Xiao, Yao Liu, Xiaoyong Qu, Logen Liu, Guo-Qing Li, Honghui Chen, Linlin Zhou, Yanping Liu

**Affiliations:** ^1^ Hunan Provincial Key Laboratory of Basic and Clinical Pharmacological Research of Gastrointestinal Cancer, Department of Gastroenterology, the Second Affiliated Hospital, Hengyang Medical School, University of South China, Hengyang, Hunan, China; ^2^ Department of Gastroenterology, the First Affiliated Hospital of Shaoyang University, Shaoyang, Hunan, China; ^3^ Department of Hepatobiliary and Pancreatic Surgery, Second Affiliated Hospital, University of South China, Hengyang, Hunan, China

**Keywords:** hepatocellular carcinoma, RNA-sequencing, aryl hydrocarbon receptors, glucose and lipid metabolism, mRNAs

## Abstract

**Background:**

Hepatocellular carcinoma (HCC) is a leading cause of cancer-related deaths with limited treatment options. Tumor metabolic disorder is elevated in HCC and activates the aryl hydrocarbon receptor (AHR), a transcription factor implicated in cancer progression. However, the role of AHR in regulating long non-coding RNAs (lncRNAs) and their impact on glycolipid metabolism remains underexplored.

**Materials and methods:**

We investigated AHR’s influence on several HCC cell lines treated with the AHR ligand. RNA sequencing was performed to identify the differentially expressed (DE) lncRNAs and mRNAs. We analyzed the differences and then conducted functional pathway enrichment of the identified DE lncRNAs and mRNAs. Furthermore, we constructed co-expression networks of lncRNAs and mRNAs and performed survival analysis using The Cancer Genome Atlas (TCGA) data.

**Results:**

RNA sequencing identified a substantial number of lncRNAs and mRNAs. DEG analysis identified the significant differences between them related to cancer progression, with pathways such as PI3K-Akt, VEGF, and PPAR signaling highlighted. A co-expression network was utilized to elucidate the lncRNA–mRNA interactions and their regulation of glycolipid metabolism.Survival analysis identified the AHR-regulated lncRNAs associated with poor prognosis, like *ASAP1-IT1* and *RMDN2-AS1*.

**Conclusion:**

This study clarifies AHR’s role in regulating gene expression and metabolism in HCC, revealing novel lncRNA biomarkers and potential therapeutic targets that could aid HCC. Further research is needed to explore AHR’s effects on the regulation of glucose-lipid metabolism in HCC.

## Introduction

Hepatocellular carcinoma (HCC) is the most prevalent form of primary liver cancer and a leading cause of cancer-related mortality worldwide (1). It often arises from chronic liver diseases, such as hepatitis B or hepatitis C infections, alcohol abuse, metabolic syndrome, or exposure to aflatoxins. One pathway connecting chronic inflammation to cancer development is via activation of the aryl hydrocarbon receptor (AHR), a transcription factor that can be stimulated by both endogenous and exogenous ligands produced during inflammatory processes (2). Inflammation induces changes in cellular metabolism, and AHR contributes to the metabolic alterations in cancer cells by regulating glycolysis and lipid metabolism through its interactions with various ligands (3).

AHR, initially recognized for its role in mediating the toxic effects of environmental pollutants like dioxins, functions as a ligand-activated transcription factor. Upon ligand binding, AHR translocates to the nucleus, where it partners with the AHR nuclear translocator to regulate gene expression by binding to dioxin or aryl hydrocarbon response elements (4, 5). While AHR’s activation has been classically associated with responses to environmental toxins, recent reports have suggested that it also plays a significant role in cancer biology (6, 7). Specifically, AHR activation in HCC has been linked to key oncogenic processes, including cellular proliferation, migration, epithelial-to-mesenchymal transition, and resistance to apoptosis (8–11).

Despite the increasing knowledge about AHR’s impact on protein-coding genes in cancer, its regulation of long non-coding RNAs (lncRNAs) in HCC remains largely unexplored. LncRNAs are critical regulators of gene expression and are now being recognized for their roles in cancer, particularly in controlling pathways involved in tumor initiation, progression, and metastasis (12, 13). However, the specific gene signatures and pathways through which AHR modulates lncRNAs in HCC have yet to be fully elucidated.

To address this gap, our study investigated how AHR activation influences the expression of both lncRNAs and mRNAs in HCC cells. Using 6-formylindolo(3, 2-b)carbazole (FICZ), a potent AHR ligand, we activated AHR in three HCC cell lines and a human fetal hepatocyte line. By combining RNA sequencing with bioinformatics analysis, we were able to identify the AHR-regulated lncRNAs and mRNAs and then explored their involvement in the glucose-lipid metabolism related pathways of HCC. This study provides new insights into the role of AHR in HCC progression and highlights lncRNAs as potential therapeutic targets and biomarkers for HCC.

## Materials and methods

### Cell culture

The human hepatocellular carcinoma (HCC) cell lines Huh7, HepG2, and SMMC-7721, and human fetal hepatocyte line LO2 were cultured in DMEM (HCC lines) or RPMI-1640 (LO2). The HCC cell lines Huh7, HepG2, and SMMC-7721 were chosen because they originate from the liver cancer tissues of patients of different ages and with different etiologies, therefore potentially representing distinct histological subtypes of HCC. This diversity allows for a broader investigation of lipid metabolic abnormalities across varying HCC contexts and enhances the generalizability of the findings.

We conducted preliminary experiments with varying the concentration of FICZ (50, 100, 200, and 400 nM) and the treatment duration (12, 24, and 48 h). We found that the cells treated with 200 nM FICZ for 24 h showed the strongest expression of the AHR target genes TIPARP and CYP1A1 based on the qPCR results, justifying the choice of this condition for further study. Cells were grown to 70%–80% confluency and treated with various concentrations of FICZ (50, 100, 200, 400 nM; n=3) for 12 or 24 h. Based on the IC50 value, treatment with 200 nM FICZ for 24 h was chosen for the further experiments. Meanwhile the control groups were treated with DMSO.

### Immunofluorescence assay

HCC or LO2 cells were cultured in 24-well dishes containing poly-L-lysine-treated coverslips. The cells were treated with either FICZ or DMSO for 24 h and then fixed with 4% paraformaldehyde (in phosphate-buffered saline, PBS) at room temperature for 15 min. After fixation, the cells were washed three times with PBS, for 5 minutes each wash. The coverslips were then permeabilized with 0.1% Triton X-100 in PBS for 15 min and subsequently blocked with 5% fetal bovine serum (FBS) to reduce non-specific binding. The cells were then incubated with a rabbit anti-AHR primary antibody (Abcam, 1:300 dilution) at 4°C overnight. Following the primary antibody incubation, the coverslips were washed three times with PBS containing 0.5% Tween 20 (PBST), for 5 min each wash. Next, cross-absorbed Alexa Fluor 488-conjugated goat anti-rabbit secondary antibody (ThermoFisher, 4 µg/mL) was then applied and the samples were incubated at room temperature for 1 h in the dark. After this secondary antibody incubation, the samples were washed three times with PBST, for 5 min each wash. Finally, the coverslips were mounted onto microscope slides using 20 μl ProLong Gold Antifade Mountant with DAPI (ThermoFisher) to stain the cell nuclei. After air drying, the samples were visualized under a fluorescence microscope.

### RNA extraction and quantification

Total RNA was extracted from the treated cells and control cells using Trizol. After lysing the cells with Trizol, chloroform was added, and the aqueous phase was collected and precipitated with isopropanol. The obtained RNA was washed with ethanol, dissolved in DEPC water, and then stored at -80°C. The RNA purity and concentration were assessed using a NanoPhotometer^®^ system and by gel electrophoresis.

### RNA sequencing library preparation and sequencing

Total RNA samples were processed for preparation of the cDNA library, with sequencing performed by BGI Group using the Illumina HiSeq platform. Eight samples (three replicates each) were sequenced, generating an average of 10.17 Gb of data per sample with an average alignment rate of 88.77%. A total of 124,841 transcripts were identified, including 12,597 novel lncRNAs, 7,264 novel mRNAs, 69,206 known lncRNAs, and 35,774 known mRNAs.

### RNA-seq alignment, annotation, and gene counting

Clean reads were aligned to the human reference genome (hg38, GRCh38) using HISAT2 (14). Transcripts were assembled with StringTie (15), and their coding potential was evaluated using CPC (16), txCdsPredict, and CNCI (17). Transcripts with coding potential and alignments with protein through Pfam-scan (18) were regarded as mRNAs; while the other transcripts were regarded as lncRNAs. The gene expression levels were quantified with RSEM. Functional annotation of the genes was performed using the GEO, Ensembl, NONCODE, and UCSC databases.

### lncRNA-mRNA co-expression network construction

Potential lncRNA targets were predicted by constructing a co-expression network of lncRNAs and mRNAs. By calculating the Pearson correlation coefficient between known annotated lncRNAs and mRNAs, lncRNA–mRNA pairs were selected based on an absolute value of the correlation ≥ 0.9, and a significance threshold of *p* < 0.05. These selected pairs were used to construct the lncRNA–mRNA co-expression network. Data visualization was performed using Cytoscape.

### Differentially expressed gene analysis

DEGs were identified using the limma package in R (19). Genes with significant expression changes (|fold change| ≥ 2, FDR ≤ 0.05) were selected. The DEG analysis included comparisons between the DMSO- and FICZ-treated groups across different cell lines. We applied a threshold fold change ≥ 2 and FDR ≤ 0.05 to maintain strong, reliable signals, as we found a lower fold-change cutoff would result in an overwhelming number of DEGs.

### Functional pathway enrichment

Gene Ontology (GO) and Kyoto Encyclopedia of Genes and Genomes (KEGG) pathway enrichment analyses were performed using the R package clusterProfiler (20).

### Quantitative reverse transcription PCR

For the reverse transcription, the RNA and primers were added to a PCR tube with a total volume of 10 µL. The mixture was incubated at 70°C for 10 min, and then quickly cooled on ice for 2 min. Subsequently, to the reaction mixture containing the RNA/primer denaturation solution, a 10 mM dNTP mixture and other reagents were added to obtain a total volume of 20 µL. The mixture was incubated at 42°C for 60 min, followed by 15 min at 72°C. The resulting cDNA was stored at -20°C for later use.

Next, the cDNA was diluted fivefold and mixed with forward and reverse primers, SYBR^®^ Premix Ex Taq™ (Tli RNaseH Plus) (2×), in a total volume of 20 µL. The PCR reaction conditions were as follows: 95°C for 5 min; 95°C for 10 s, followed by 60°C for 34 s (when the fluorescence signal was collected), for 39 cycles. Melt curve analysis was performed by heating the mixture from 60°C to 95°C, with fluorescence measurements taken every minute.

The reaction mixture was loaded into a 96-well PCR plate and centrifuged to ensure the contents were well-settled at the bottom. The reactions were conducted using a Bio-Rad CFX96 real-time PCR system under the following conditions: 95°C for 2 min; 43 cycles at 95°C for 15 s; 58°C for 5 s; and 72°C for 20 s. Fluorescence data were collected at the 72°C step for each cycle.

The primers were designed using PRIMER5 software or the NCBI online tool and are listed in **Table 1**.

**Table 1:** PCR primer sequences.

Gene	Sequence	Product size(bp)
lnc ASAP1-IT1	F:5’-AAACATCATCCCCAGAGTGG-3’	147
lnc ASAP1-IT1	R:5’- GCCTTGCTCACCTCTGAAAC-3’	
NONHSAT221345.1*	F:5’-TCTCTGTTGGCTGGTGCAAT-3’	97
(NON345)#	R:5’-TGCTTTCGGCACAGAGTCAT-3’	
lnc-TP53TG5-6	F:5’-CGGCTGCGTAGGAAAGAAAC-3’	104
lnc-TP53TG5-6	R:5’-CTATCCGGCTGCTTGTACCT-3’	
lnc-TMEM232-4	F:5’-CCACTATGGTGCATTTGATCCT-3’	159
lnc-TMEM232-4	R:5’-GCTTCCATTTACTGTGTGTGTCC-3’	
RMDN2-AS1	F:5’-TTCCTCTTTTGTGCTGCTTCTC-3’	116
RMDN2-AS1	R:5’-GTACCGCAAGCCCTGTCATC-3’	
lnc-DGKK-1	F:5’-TGACACCACAGCTTTCCTGG-3’	168
lnc-DGKK-1	R:5’-TATTCATGGCATCCAGGGCG-3’	
lnc-FAM237B-2	F:5’-AGGACCCGAAGTACCGAACA-3’	201
lnc-FAM237B-2	R:5’-CATGCTTTGACGCTGGTAGT-3’	
lnc-FGA-2	F:5’-TGTCCAACTACCTGTGGCAT-3’	125
lnc-FGA-2	R:5’-ACAACAGCAAAAGAACTTCACA-3’	
DIPK1B	F:5’-GTGCTCTTCTGCCCCTTCTC-3’	184
DIPK1B	R:5’-TGCGGTACTGGTCACAAATGA-3’	
BBC3	F:5’-GAAGGACAAAACTCACCAAACCA-3’	187
BBC3	R:5’-GCTCCCTGGGGCCACAAA-3’	
DEFB1	F:5’-AGATGGCCTCAGGTGGTAAC-3’	100
DEFB1	R:5’-GGGCAGGCAGAATAGAGACA-3’	
IGFBP3	F:5’-GCCAGCTCCAGGAAATGCTA-3’	109
IGFBP3	R:5’-GGGGTGGAACTTGGGATCAG-3’	
CPA4	F:5’-AGGACCTGCAGATTTACCACG-3’	98
CPA4	R:5’-CGGCCGGTTTTCAAACGAAT-3’	
RhoBTB1	F:5’-CGGCTTCAGGGTAAGTCCAG-3’	208
RhoBTB1	R:5’-AGCAGCTGATACTGCGTGAG-3’	
ANKRD1	F:5’-ACAAGTGGACACTCGCAGTC-3’	142
ANKRD1	R:5’-CCCTGCTGAACAAGCCAAAC-3’	
ANPEP	F:5’-TGGCCACTACACAGATGCAG-3’	145
ANPEP	R:5’-CTGGGACCTTTGGGAAGCAT-3’	
ASAP1	F:5’-CGGTCGCAGTTCGCTTTCC-3’	108
ASAP1	R:5’-GCACAGGGAGGCCAACAC-3’	
CYP1A1	F:5’-TCAGTACCTCAGCCACCTCC -3’	169
CYP1A1	R:5’-CATGGCCCTGGTGGATTCTT -3’	
TIPARP	F:5’-GGTCGAGGCTTTCTGCGTTC-3’	250
TIPARP	R:5’-GCACTACACAGTCTGGCTCA-3’	
GAPDH-F	F:5’-GGTGGTCTCCTCTGACTTCAA-3’	258
GAPDH-R	R:5’-GTTGCTGTAGCCAAATTCGTTGT-3’	
β-actin-F	F:5’-CTCCATCCTGGCCTCGCTGT-3’	268
β-actin-R	R:5’-GCTGTCACCTTCACCGTTCC-3’	

bp, base pair; *NONCODE transcript ID, #abbreviation.

### Survival analysis

Survival analysis was conducted using data from The Cancer Genome Atlas (TCGA). The expression levels of the selected lncRNAs were correlated with the patient survival outcomes. Statistical analysis was used to determine the prognostic significance of these lncRNAs, for identifying potential biomarkers for liver cancer prognosis.

### Statistical analysis

All the data were statistically analyzed using SPSS software (version 13.0, SPSS Inc., Chicago, IL, USA), GraphPad Prism 8.0, or R. One-way analysis of variance (ANOVA) was performed, followed by unpaired t-tests where appropriate. A p-value < 0.05 was considered statistically significant. Using the survival package in R, we performed a proportional hazards assumption test and Cox regression analysis on Overall Survival (OS) using liver cancer data from the TCGA database (GDC Portal).

## Results

### Transcriptome profiling of the mRNAs and lncRNAs after AHR activation in HCC cells

To study the role of AHR in liver cancer, we induced AHR activation in HCC cells using 200 nM FICZ. We selected three hepatocellular carcinoma cell lines (HepG2, Huh7, and SMMC-7721) that have enriched plasma unactivated AHR, as well as the human fetal hepatocyte line LO2. We treated these cells with FICZ, creating four FICZ-treatment groups, as well as four DMSO control groups for comparison.

**Figure 1:**
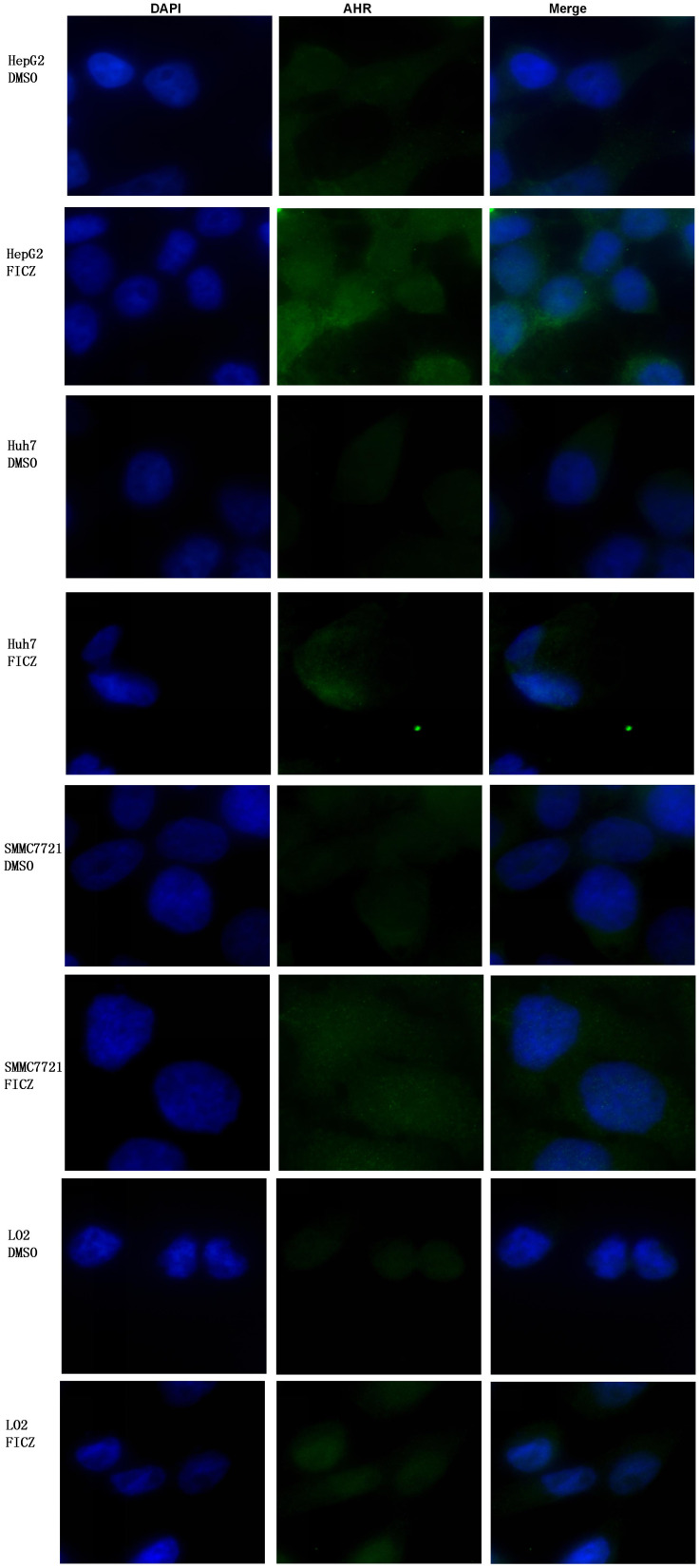
Comparison of AHR nuclear translocation between three liver cancer cell lines and LO2 before and after FICZ treatment: HepG2-FICZ vs. HepG2-DMSO, Huh7-FICZ vs. Huh7-DMSO, SMMC7721-FICZ vs. SMMC7721-DMSO, and LO2-FICZ vs. LO2-DMSO.

We assessed AHR activation and nuclear translocation in the liver cancer cell lines after FICZ treatment using immunofluorescence assays. The results showed a significant increase in AHR fluorescence within the nuclei of the FICZ-treated cells. In contrast, the DMSO-treated controls displayed no such nuclear fluorescence, confirming that FICZ treatment successfully activated AHR, as indicated by the strong fluorescence burst seen in both the nucleus and cytoplasm, while the control DMSO group showed only weak AHR fluorescence, primarily localized in the cytoplasm (**Figure 1**).

**Figure 2:**
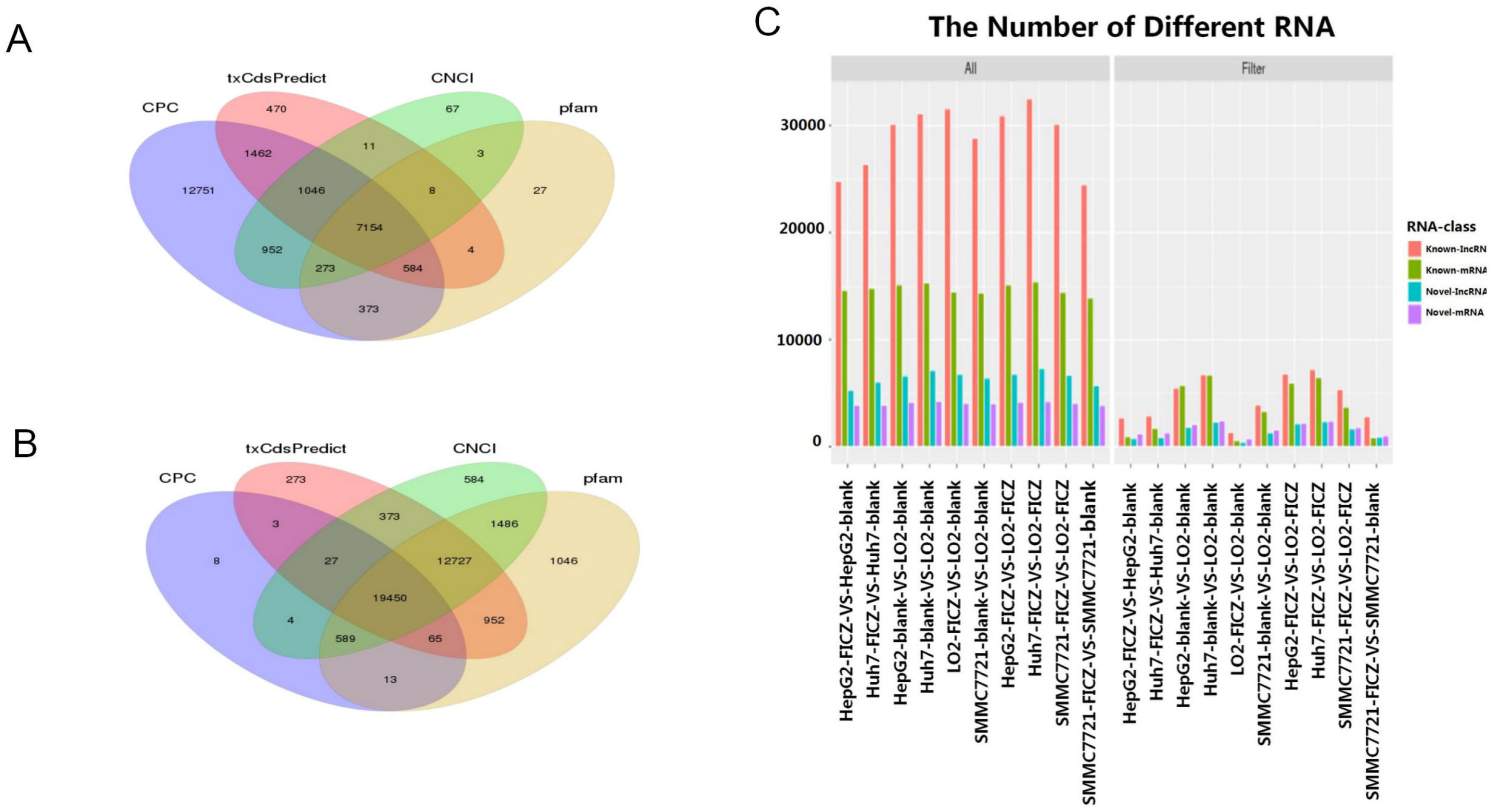
Annotation of the mRNA and lncRNA transcripts. **(A, B)** Venn diagram showing the transcript coding potential predictions by various methods. **(C)** Differences in the captured transcripts of the tumor cell line FICZ-treated groups compared to the DMSO control groups, as well as the normal cell line LO2 groups.

Next, we performed RNA-seq to analyze the transcriptomic changes induced by AHR activation, with the workflow customized to analyze both mRNAs and lncRNAs. Given our interest in both mRNAs and long non-coding RNAs (lncRNAs), we used StringTie for *de novo* transcript assembly, achieving an average genome alignment rate of 88.77%. We classified the transcripts as mRNAs if they were predicted to have coding potential by at least three of the following four methods: txCdsPredict, CNCI, Pfam-scan, and CPC (**Figure 2A**). Conversely, transcripts that were identified as non-coding by at least three of these methods were classified as lncRNAs (**Figure 2B**).

We then cross-referenced these transcripts with annotated databases to distinguish between annotated and novel mRNAs and lncRNAs. In total, we identified 124,841 transcripts, comprising 69,206 annotated lncRNAs, 12,597 novel lncRNAs, 35,774 annotated mRNAs, and 7,264 novel mRNAs. The distribution of these transcript categories was consistent across the different cell lines (**Figure 2C**).

### AHR activation alters both coding and non-coding gene expressions in HCC cells

**Figure 3:**
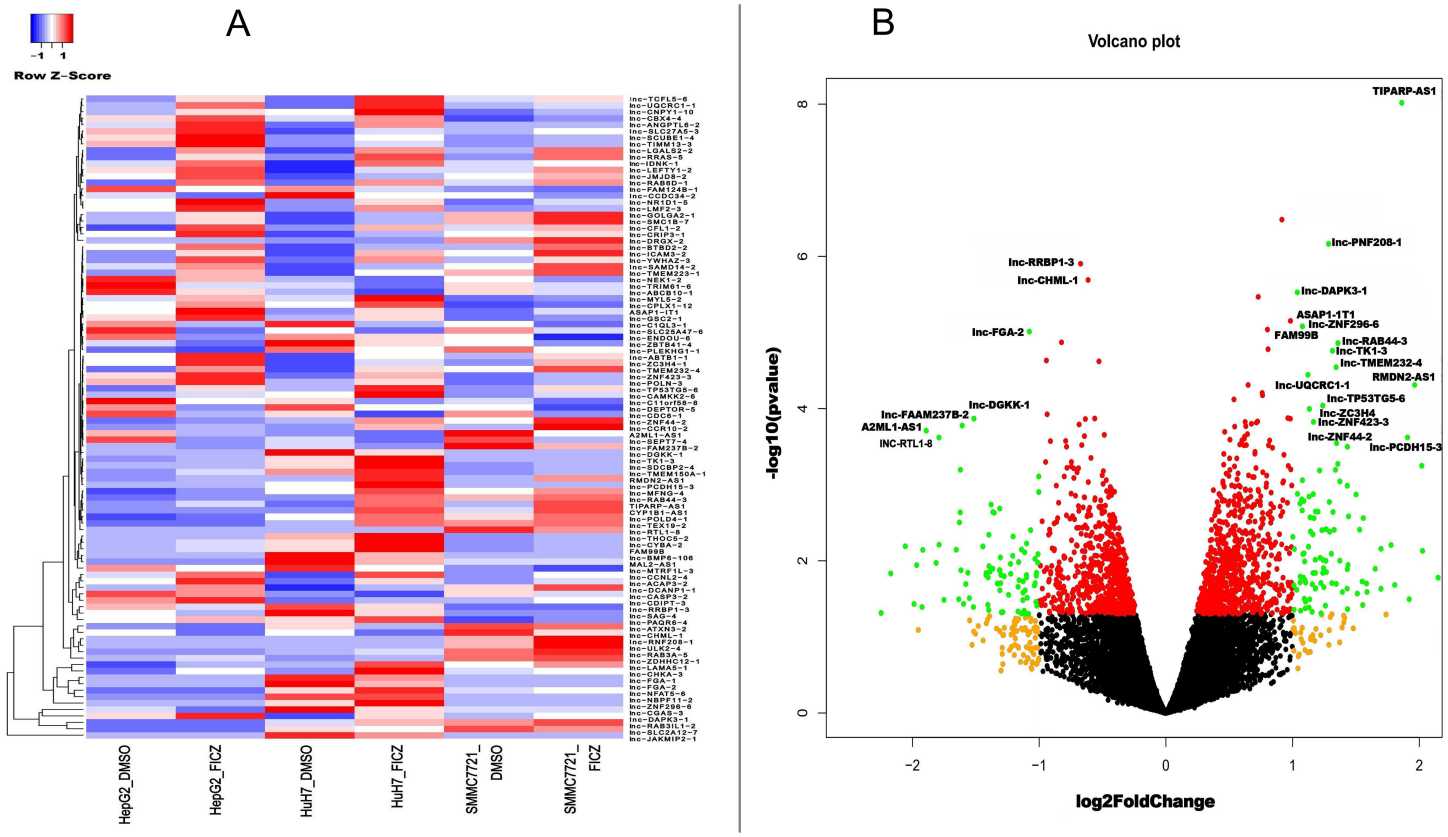
DE lncRNAs after AHR activation. **(A)** Heatmaps showing the average expressions of the DE lncRNAs. **(B)** Volcano plot indicating the up- and downregulated lncRNAs after AHR activation.|log2Fold Change|≥ 1, FDR < 0.05.

**Figure 4:**
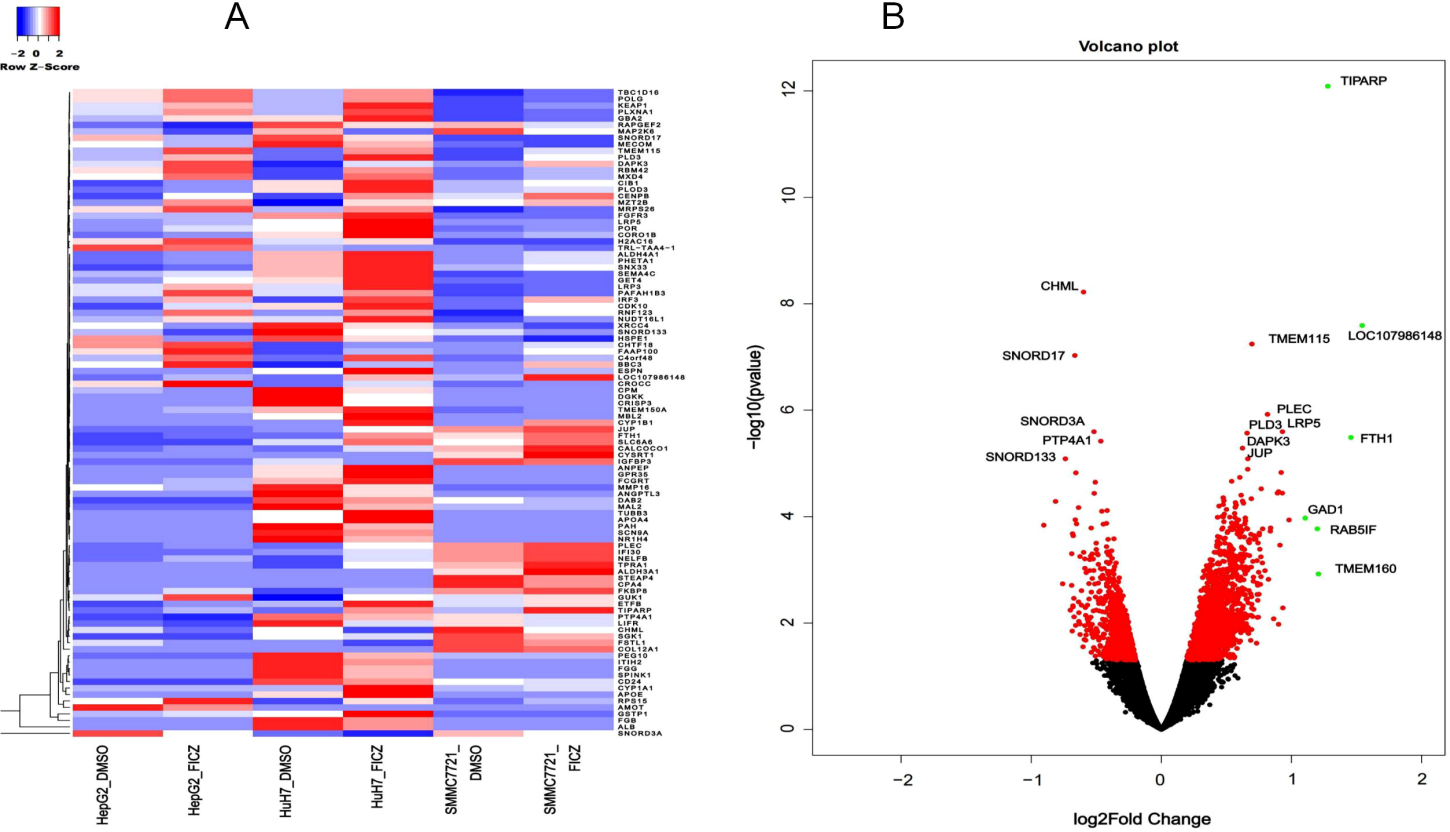
DE mRNAs after AHR activation. **(A)** Heatmaps showing the average expression of the DE RNAs **(B)** Volcano plot indicating the up- and downregulated mRNAs after AHR activation.|log2Fold Change|≥ 1, FDR < 0.05.

To further investigate the common AHR target genes in the tested HCC cells, we conducted differential gene expression (DEG) analysis by comparing tumor cell lines with or without AHR activation. Additionally, we included a control group based on a human fetal hepatocyte line to filter out genes that were altered upon AHR activation in non-cancerous conditions. This approach allowed us to focus specifically on AHR-related changes in cancer cells. Our analysis identified 427 significantly differentially expressed lncRNA transcripts with known gene annotations, of which 167 (39.1%) were upregulated and 260 (60.9%) were downregulated (**Table 2**, **Figures 3A, B**). We also found 413 significantly differentially expressed mRNA transcripts, with 321 (77.7%) upregulated and 92 (22.3%) downregulated (**Table 3**, **Figures 4A, B**). This analysis revealed that AHR exerts broad regulatory effects on both non-coding and coding genes, with a common gene expression signature associated with liver cancer.

**Table 2:** Significantly up- and downregulated lncRNAs in hepatocellular carcinoma cells.

Genes	Symbol	logFC	logCPM	LR	P-value	FDR
778	TIPARP-AS1	1.861620117	3.214737821	32.93799673	9.51E-09	0.00011269
8294	lnc-RAB6D-1	0.915705342	4.13637338	26.08612214	3.27E-07	0.001933666
10979	lnc-ULK2-4	1.284448784	4.154834709	24.66664525	6.82E-07	0.002690702
8793	lnc-RRBP1-3	-0.673220386	5.580449407	23.48582645	1.26E-06	0.003726045
2644	lnc-CHML-1	-0.613606784	5.385465695	22.5321236	2.07E-06	0.004895331
8641	lnc-RNF208-1	1.038390722	4.534862621	21.8257067	2.99E-06	0.005704803
3190	lnc-DAPK3-1	0.728946943	7.512629532	21.59252526	3.37E-06	0.005704803
11562	lnc-ZNF296-6	1.079771101	6.26686713	19.84960719	8.38E-06	0.010538446
86	CYP1B1-AS1	2.615309967	2.735214951	19.7043596	9.04E-06	0.010538446
149	FAM99B	0.801612343	3.679611353	19.68519978	9.13E-06	0.010538446
**4221**	**lnc-FGA-2**	**-1.077423142**	**5.357748258**	**19.55245659**	**9.79E-06**	**0.010538446**
1778	lnc-BMP6-106	-0.823554772	3.912233937	18.93229175	1.35E-05	0.012483127
8289	lnc-RAB44-3	1.358001043	3.005330609	18.91024217	1.37E-05	0.012483127
8981	lnc-SCUBE1-4	0.806428823	3.92812318	18.55794394	1.65E-05	0.013688554
10310	lnc-TK1-3	1.3150673	2.331048875	18.46161935	1.73E-05	0.013688554
5430	lnc-JAKMIP2-1	-0.942690624	7.358205779	17.88286954	2.35E-05	0.016818688
3808	lnc-EPN2-3	-0.527975223	12.0157068	17.83111678	2.41E-05	0.016818688
**10461**	**lnc-TMEM232-4**	**1.346600815**	**1.657119171**	**17.53223729**	**2.82E-05**	**0.018587009**
11014	lnc-UQCRC1-1	1.121289986	4.536438213	17.08894942	3.57E-05	0.022235083
**670**	**RMDN2-AS1**	**1.963610682**	**2.281631062**	**16.50268569**	**4.86E-05**	**0.027518695**
10291	lnc-TIMM13-3	0.649245906	4.236970446	16.49447639	4.88E-05	0.027518695
5444	lnc-JMJD8-2	0.760220917	3.729611642	16.03905478	6.20E-05	0.033405069
2346	lnc-CCNL2-4	0.762903105	5.393368095	15.90688047	6.65E-05	0.03426335
3219	lnc-DCANP1-1	0.537327093	5.231107145	15.67187408	7.53E-05	0.037178404
**10620**	**lnc-TP53TG5-6**	**1.238249295**	**1.333157722**	**15.30802125**	**9.13E-05**	**0.043267501**
11415	lnc-ZC3H4-1	1.132244197	1.605069144	15.11757456	0.000101018	0.046017797
2422	lnc-CDC6-1	-0.936850787	2.079025026	14.79604461	0.000119786	0.049646064
5167	lnc-ICAM3-2	0.96279571	2.328240638	14.59194882	0.000133484	0.049646064
6577	lnc-MTRF1L-3	-0.561597343	4.864436688	14.56122465	0.000135678	0.049646064
6623	lnc-MYL5-2	0.982192184	2.553897541	14.55803027	0.000135908	0.049646064
**3328**	**lnc-DGKK-1**	**-1.514802777**	**2.16163716**	**14.55710583**	**0.000135975**	**0.049646064**
3972	lnc-FAM124B-1	-0.635278868	3.834562201	14.52598851	0.000138239	0.049646064
1274	lnc-ANGPTL6-2	0.723893031	4.533359082	14.41688161	0.000146483	0.049646064
8792	lnc-RRAS-5	0.625562467	3.685629613	14.38388709	0.000149072	0.049646064
11607	lnc-ZNF423-3	1.164801851	1.830057644	14.37737352	0.000149589	0.049646064
3145	lnc-CYBA-2	0.714322884	4.104690663	14.34650505	0.000152062	0.049646064
7038	lnc-NR1D1-5	0.675420448	3.406753676	14.27452805	0.000157988	0.049646064
1950	lnc-C1QL3-1	-0.684587682	3.302935093	14.21665185	0.000162922	0.049646064
**4069**	**lnc-FAM237B-2**	**-1.607543135**	**0.99066209**	**14.16617456**	**0.000167352**	**0.049646064**
11438	lnc-ZDHHC12-1	0.631017126	5.850149956	14.14649509	0.000169112	0.049646064
9529	lnc-SMC1B-7	0.543249504	4.483341944	14.11619015	0.000171858	0.049646064
**29065**	**ASAP1-IT1**	**1.522478901**	**3.322431032**	**13.37489082**	**0.000162893**	**0.049646064**
NA*	**NONHSAT221345.1**	**2.143172122**	**1.965842132**	**14.1568425**	**0.000472748**	**0.000534819**

Gene symbols in bold font indicate those selected for qPCR validation. logFC, Log fold change; logCPM, Log counts per million; LR, Likelihood ratio; FDR, False discovery rate.

*NONHSAT221345.1 is not present in the NCBI database; therefore, a gene ID is not available.

**Table 3:** Significantly up- and downregulated mRNAs in hepatocellular carcinoma cells.

Genes	logFC	logCPM	LR	P-value	FDR	Gene	Annotation
**5670**	**4.09368978**	**7.846445625**	**53.62467018**	**2.43E-13**	**2.26E-09**	**CYP1A1**	**cytochrome P450 family 1 subfamily A member 1**
**7636**	**1.331519163**	**6.787145918**	**52.85478682**	**3.59E-13**	**2.26E-09**	**TIPARP**	**TCDD inducible poly(ADP-ribose) polymerase**
5394	3.359394478	4.725310823	48.61531296	3.11E-12	1.31E-08	CYP1B1	cytochrome P450 family 1 subfamily B member 1
2634	1.217573006	5.470930279	45.78358675	1.32E-11	4.16E-08	ALDH3A1	aldehyde dehydrogenase 3 family member A1
11055	-0.601320829	7.172574898	32.09635631	1.47E-08	3.70E-05	CHML	CHM like, Rab escort protein 2
9702	0.942898234	6.626305252	31.59570812	1.90E-08	3.99E-05	HIST3H2A	histone cluster 3 H2A
**4320**	**-1.180896231**	**5.447437596**	**28.17149231**	**1.11E-07**	**0.000199898**	**CPA4**	**carboxypeptidase A4**
11283	-0.667621379	5.689874004	27.10692861	1.93E-07	0.00030327	SNORD17	small nucleolar RNA, C/D box 17
11981	-0.684425946	8.093246442	25.76426705	3.86E-07	0.000540199	PEG10	paternally expressed 10
8334	1.262492876	5.260448017	25.34982457	4.78E-07	0.00060267	FTH1	ferritin heavy chain 1
9472	0.819163977	6.570565036	24.13064168	9.00E-07	0.001031344	PLEC	plectin
7427	0.930269587	6.013558309	22.44063441	2.17E-06	0.002276309	LRP5	LDL receptor related protein 5
2260	0.657432682	5.154227466	21.4463086	3.64E-06	0.003503863	PLD3	phospholipase D family member 3
2212	0.919706005	4.463913651	21.31719035	3.89E-06	0.003503863	FCGRT	Fc fragment of IgG receptor and transporter
4174	0.645208779	4.859350648	20.94816109	4.72E-06	0.003964741	TMEM115	transmembrane protein 115
12005	1.612486369	1.705016314	20.37433378	6.37E-06	0.004978437	C4orf48	chromosome 4 open reading frame 48
9052	0.655363176	5.258989178	20.27265899	6.72E-06	0.004978437	JUP	junction plakoglobin
8494	0.584468534	6.989177507	20.02891802	7.63E-06	0.00534086	SLC25A6	solute carrier family 25 member 6
**8161**	**1.095602399**	**3.90335853**	**19.61968386**	**9.45E-06**	**0.006267717**	**ANPEP**	**alanyl aminopeptidase, membrane**
8275	0.603265455	5.157896505	19.4547261	1.03E-05	0.006464857	DAPK3	death associated protein kinase 3
2962	-0.454963333	7.179414015	19.36935315	1.08E-05	0.006464857	PTP4A1	protein tyrosine phosphatase type IVA, member 1
11334	0.807102047	4.051601191	18.84937289	1.41E-05	0.008103592	RPLP0P6	ribosomal protein lateral stalk subunit P0 pseudogene 6
8365	0.914452466	6.153657685	18.40206891	1.79E-05	0.009800952	DDIT4	DNA damage inducible transcript 4
10844	0.664658442	4.453101429	18.2332433	1.95E-05	0.010262924	PHETA1	PH domain containing endocytic trafficking adaptor 1
7673	0.787013747	6.017910668	18.13673165	2.06E-05	0.010364554	TPRA1	transmembrane protein adipocyte associated 1
10459	-0.858231332	3.367315275	17.87173142	2.36E-05	0.011226427	NEMP2	nuclear envelope integral membrane protein 2
6943	-0.905659928	3.850836333	17.78066575	2.48E-05	0.011226427	MMP16	matrix metallopeptidase 16
3963	0.888251009	5.485211937	17.76897988	2.49E-05	0.011226427	MXD4	MAX dimerization protein 4
1225	0.798927854	9.682476156	17.66958351	2.63E-05	0.01142073	GSTP1	glutathione S-transferase pi 1
3543	-0.50454512	7.087458312	17.52465945	2.84E-05	0.011679981	SGK1	serum/glucocorticoid regulated kinase 1
**6264**	**-1.005814301**	**4.296796517**	**17.47451716**	**2.91E-05**	**0.011679981**	**IGFBP3**	**insulin like growth factor binding protein 3**
3082	-0.513677706	6.914625767	17.43971932	2.97E-05	0.011679981	LIFR	LIF receptor alpha
**7998**	**1.112559395**	**3.67916475**	**16.87025046**	**4.00E-05**	**0.01491383**	**DIPK1B**	**divergent protein kinase domain 1B**
10727	-0.677616662	6.98369926	16.82050363	4.11E-05	0.01491383	DPP4	dipeptidyl peptidase 4
6002	0.632020996	6.832046788	16.80530092	4.14E-05	0.01491383	GUK1	guanylate kinase 1
3228	0.501224167	8.649564254	16.39368843	5.15E-05	0.017571652	RPS15	ribosomal protein S15
8224	0.488219003	5.684882134	16.38887909	5.16E-05	0.017571652	TBC1D16	TBC1 domain family member 16
2431	0.578835666	5.158057976	16.17677656	5.77E-05	0.01840022	PLOD3	procollagen-lysine,2-oxoglutarate 5-dioxygenase 3
5639	0.47995143	7.099569319	16.16522145	5.81E-05	0.01840022	SERF2	small EDRK-rich factor 2
**2276**	**1.160969037**	**1.785853658**	**16.1470113**	**5.86E-05**	**0.01840022**	**BBC3**	**BCL2 binding component 3**
9525	0.559031503	5.730574201	16.06525767	6.12E-05	0.01840022	GAK	cyclin G associated kinase
3158	0.527599033	6.04683465	16.06145751	6.13E-05	0.01840022	PLXNA1	plexin A1
4195	0.583057023	4.936443432	15.93076601	6.57E-05	0.019256802	IRF3	interferon regulatory factor 3
777	0.574213593	6.162504807	15.85772667	6.83E-05	0.019559611	FGFR3	fibroblast growth factor receptor 3
6511	0.752962433	3.859417229	15.73569928	7.28E-05	0.020399071	TM7SF2	transmembrane 7 superfamily member 2
2335	0.495367513	6.918939426	15.62342073	7.73E-05	0.021176141	FKBP8	FK506 binding protein 8
6740	-0.58364203	4.993583145	15.50891391	8.21E-05	0.021686747	DAB2	DAB2, clathrin adaptor protein
6349	-0.75115404	4.633135208	15.49791036	8.26E-05	0.021686747	MAL2	mal, T cell differentiation protein 2
8927	0.562509536	5.988557378	15.42270506	8.59E-05	0.02210653	CORO1B	coronin 1B
4155	0.441756066	6.365863921	15.27534546	9.29E-05	0.02342178	MRPS26	mitochondrial ribosomal protein S26
10856	0.533172892	6.38010935	14.90772012	0.0001129	0.027899586	RUVBL2	RuvB like AAA ATPase 2
2911	-0.660218834	7.875218468	14.85790304	0.000115921	0.02809532	COL12A1	collagen type XII alpha 1 chain
6685	-0.703274786	3.738262398	14.70374573	0.000125796	0.029716569	XRCC4	X-ray repair cross complementing 4
4188	0.561887892	5.525491986	14.68095426	0.000127326	0.029716569	RBM42	RNA binding motif protein 42
9027	0.556254762	4.64411185	14.59955618	0.000132946	0.030463937	SNX33	sorting nexin 33
4173	-0.517446716	8.303450251	14.48219614	0.000141491	0.031206038	AMOT	angiomotin
4287	0.545666095	6.035809303	14.43101265	0.000145388	0.031206038	POR	cytochrome p450 oxidoreductase
5063	-0.816119295	3.120987413	14.41181961	0.000146878	0.031206038	CPM	carboxypeptidase M
4132	0.429221773	6.506995911	14.30272458	0.000155639	0.031206038	CENPB	centromere protein B
10377	0.636737474	6.083311252	14.29168354	0.000156555	0.031206038	H2AFX	H2A histone family member X
8537	-0.735792209	4.967650717	14.27747817	0.000157741	0.031206038	SCN9A	sodium voltage-gated channel alpha subunit 9
4269	0.595332927	4.698432992	14.27524388	0.000157928	0.031206038	CHTF18	chromosome transmission fidelity factor 18
1246	-0.456968437	5.577153467	14.27114073	0.000158273	0.031206038	MECOM	MDS1 and EVI1 complex locus
3515	0.557019123	5.582251041	14.24133236	0.0001608	0.031206038	STK11	serine/threonine kinase 11
5162	0.690575097	4.367057881	14.23962978	0.000160945	0.031206038	TTYH3	tweety family member 3
4548	0.629521527	4.837311858	14.19092587	0.000165165	0.031244991	LRP3	LDL receptor related protein 3
**6427**	**-0.760171603**	**5.757128562**	**14.1802548**	**0.000166104**	**0.031244991**	**ANKRD1**	**ankyrin repeat domain 1**
7584	-0.401420968	7.84714578	14.07476322	0.000175685	0.03222134	FSTL1	follistatin like 1
8465	0.890522208	3.004186978	14.05290039	0.00017774	0.03222134	TMEM150A	transmembrane protein 150A
1123	0.477043528	5.822548406	14.00438394	0.000182385	0.03222134	KEAP1	kelch like ECH associated protein 1
7166	0.619257059	6.762889747	14.00209624	0.000182607	0.03222134	FBXW5	F-box and WD repeat domain containing 5
5432	-0.736501298	4.515286323	13.98254276	0.000184516	0.03222134	AOX1	aldehyde oxidase 1
5811	0.80533639	3.831986446	13.96107658	0.000186635	0.03222134	SLC39A3	solute carrier family 39 member 3
585	0.890389364	2.842672097	13.90594308	0.00019219	0.032510986	CROCC	ciliary rootlet coiled-coil, rootletin
9280	0.511594521	5.967602942	13.84508946	0.000198515	0.032510986	SSNA1	SS nuclear autoantigen 1
233	0.55094641	4.755208957	13.83944721	0.000199112	0.032510986	CALCOCO1	calcium binding and coiled-coil domain 1
1682	0.536403704	6.529975173	13.83247283	0.000199852	0.032510986	SBF1	SET binding factor 1
10706	-0.431613183	6.118478445	13.80086678	0.000203242	0.032510986	RPF2	ribosome production factor 2 homolog
4128	0.983713198	2.308566413	13.78826186	0.000204611	0.032510986	SDCBP2	syndecan binding protein 2
8515	0.656748109	4.777594925	13.75159398	0.000208644	0.032510986	PRELID1	PRELI domain containing 1
2729	-0.421730846	5.91335866	13.7488433	0.000208949	0.032510986	RAPGEF2	Rap guanine nucleotide exchange factor 2
3283	0.447684219	6.18297209	13.72344162	0.000211794	0.032551769	STK25	serine/threonine kinase 25
7728	0.579147638	4.153231858	13.68178745	0.000216544	0.032713944	RNF123	ring finger protein 123
7349	0.477920967	7.506243269	13.65988343	0.000219085	0.032713944	SQSTM1	sequestosome 1
10422	0.462273611	6.278359659	13.62673545	0.000222987	0.032713944	NELFB	negative elongation factor complex member B
8276	0.42858008	10.61193707	13.59137394	0.000227227	0.032713944	EEF2	eukaryotic translation elongation factor 2
8563	0.66948375	4.724134286	13.59036534	0.000227349	0.032713944	LRRC45	leucine rich repeat containing 45
6855	-0.504433822	5.290380028	13.57636043	0.000229052	0.032713944	SKA1	spindle and kinetochore associated complex subunit 1
2176	0.945338958	3.766251374	13.56030401	0.00023102	0.032713944	GSDMD	gasdermin D
5042	-0.45341215	5.545245097	13.4871124	0.000240208	0.033637066	LTV1	LTV1 ribosome biogenesis factor
1544	0.591582091	5.762046548	13.39389164	0.000252445	0.034962255	NUBP2	nucleotide binding protein 2
**7859**	**-1.306100601**	**3.780898184**	**13.35213097**	**0.000258129**	**0.035236777**	**DEFB1**	**defensin beta 1**
640	1.79714468	3.275578596	13.2965143	0.0002659	0.035236777	AHRR	aryl-hydrocarbon receptor repressor
2219	0.509192594	5.278762403	13.29289145	0.000266414	0.035236777	PLEKHJ1	pleckstrin homology domain containing J1
10551	0.821634121	3.898166594	13.28537938	0.000267484	0.035236777	CACNA1H	calcium voltage-gated channel subunit alpha1 H
592	0.591301259	4.789001504	13.27892208	0.000268407	0.035236777	FLYWCH1	FLYWCH-type zinc finger 1
4984	0.952342673	2.541906847	13.24401016	0.000273452	0.035529061	CCNJL	cyclin J like
9152	-0.832699257	4.575780177	13.20933605	0.000278558	0.035748741	PDZK1	PDZ domain containing 1
1112	0.542808377	4.72550728	13.19420498	0.000280816	0.035748741	PAFAH1B3	platelet activating factor acetylhydrolase 1b catalytic subunit 3
3131	0.946447121	3.982431272	13.15113346	0.000287345	0.036053067	PFKFB4	6-phosphofructo-2-kinase/fructose-2,6-biphosphatase 4
**50807**	**1.463104224**	**3.152643352**	**13.25351257**	**0.000561426**	**0.035237352**	**ASAP1**	**arf-GAP with SH3 domain, ANK repeat and PH domain-containing protein 1 isoform 1**
**9886**	**-1.194114467**	**7.412662654**	**13.263851**	**0.000265261**	**0.035486122**	**RhoBTB1**	**Rho related BTB domain containing 1**

Gene symbols in bold font indicate those selected for qPCR validation. logFC, Log fold change; logCPM, Log counts per million; LR, Likelihood ratio; FDR, False discovery rate.

### Validation of the differentially expressed lncRNAs and mRNAs in Huh7 cells

Next, we validated the RNA-seq findings by performing qRT-PCR on selected differentially expressed genes. We also included two well-characterized AHR target genes (*TIPARP* and *CYP1A1*) as positive controls. We selected the top 10 differentially expressed mRNAs and lncRNAs based on their absolute log fold change values and proceeded with those for which high-fidelity primers could be reliably designed. Consequently, 8 lncRNAs (*ASAP1-IT1*, *NONHSAT221345.1*, *RMDN2-AS1*, *lnc-TMEM232-4*, *lnc-TP53TG5-6*, *lnc-FGA-2*, *lnc-DGKK-1*, and *lnc-FAM237B-2*), and 9 mRNAs (*BBC3*, *ANPEP*, *DIPK1B*, *ASAP1*, *RhoBTB1*, *CPA4*, *ANKRD1*, *IGFBP3*, and *DEFB1*) were selected for validation.

**Figure 5:**
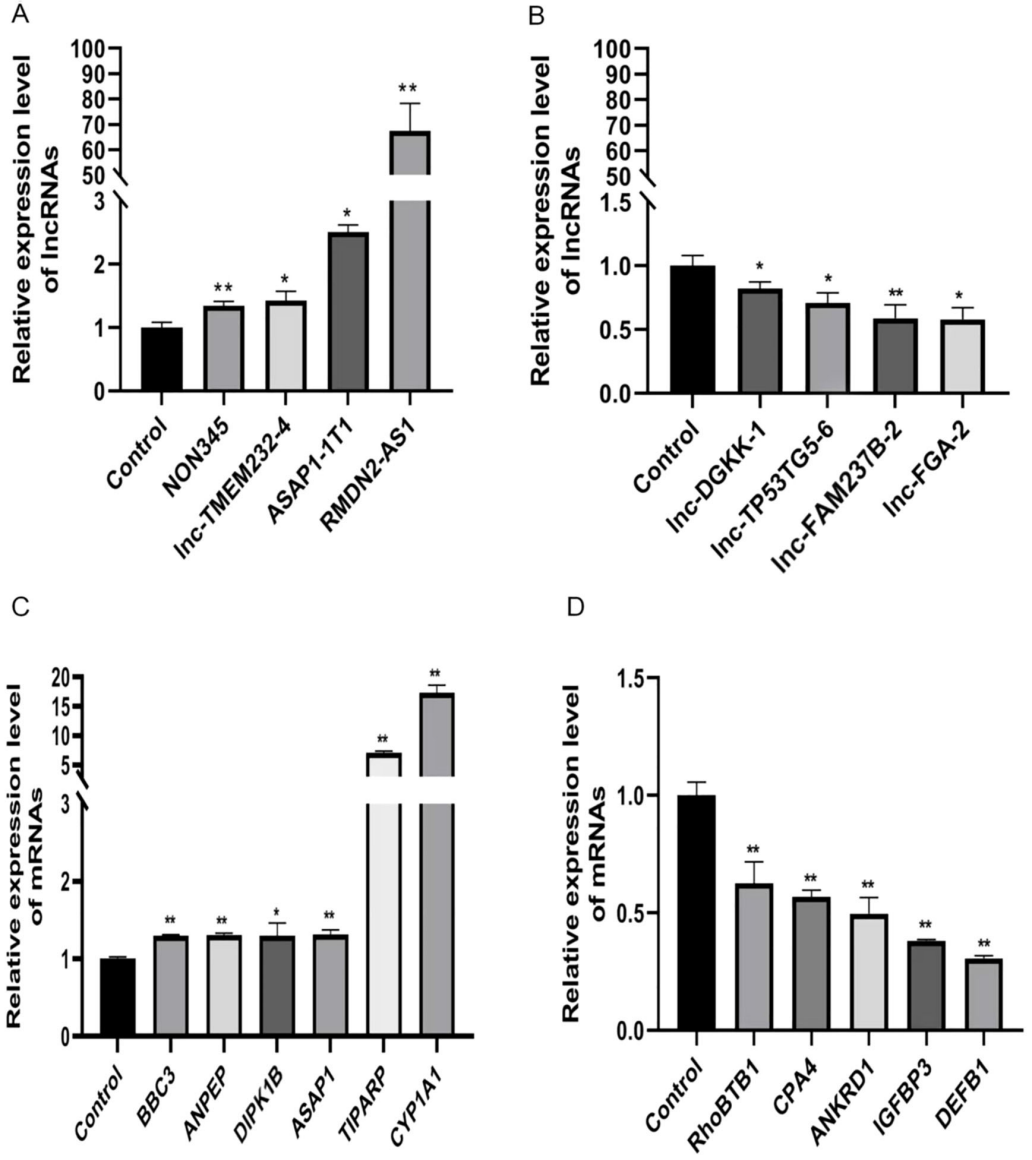
qRT-PCR validations of four dysregulated lncRNAs and mRNAs. **(A)** ASAP1-IT1, NONHSAT221345.1, RMDN2-AS1, and lnc-TMEM232-4 are four upregulated lncRNAs, **(B)** lnc-TP53TG5-6, lnc-FGA-2, lnc-DGKK-1, and lnc-FAM237B-2 are four downregulated lncRNAs. *p < 0.05, **p < 0.01. **(C)** BBC3, ANPEP, DIPK1B, ASAP1, TIPARP, and CYP1A1 are six upregulated mRNAs, **(D)** RhoBTB1, CPA4, ANKRD1, IGFBP3 and DEFB1 are five downregulated mRNAs. *p < 0.05, **p < 0.01.

We conducted qRT-PCR on Huh7 cells treated with either FICZ or DMSO. The qRT-PCR results demonstrated that, except for lnc-TP53TG5-6, the expression trends for both the lncRNAs and mRNAs in the FICZ-treated cells were consistent with those identified in the RNA-seq analysis (**Figure 5**). This confirmation supported the reliability of our high-throughput sequencing data and verified the accuracy of the observed gene expression changes.

### Functional pathways of the AHR-activation-responsive genes in HCC cells

**Figure 6:**
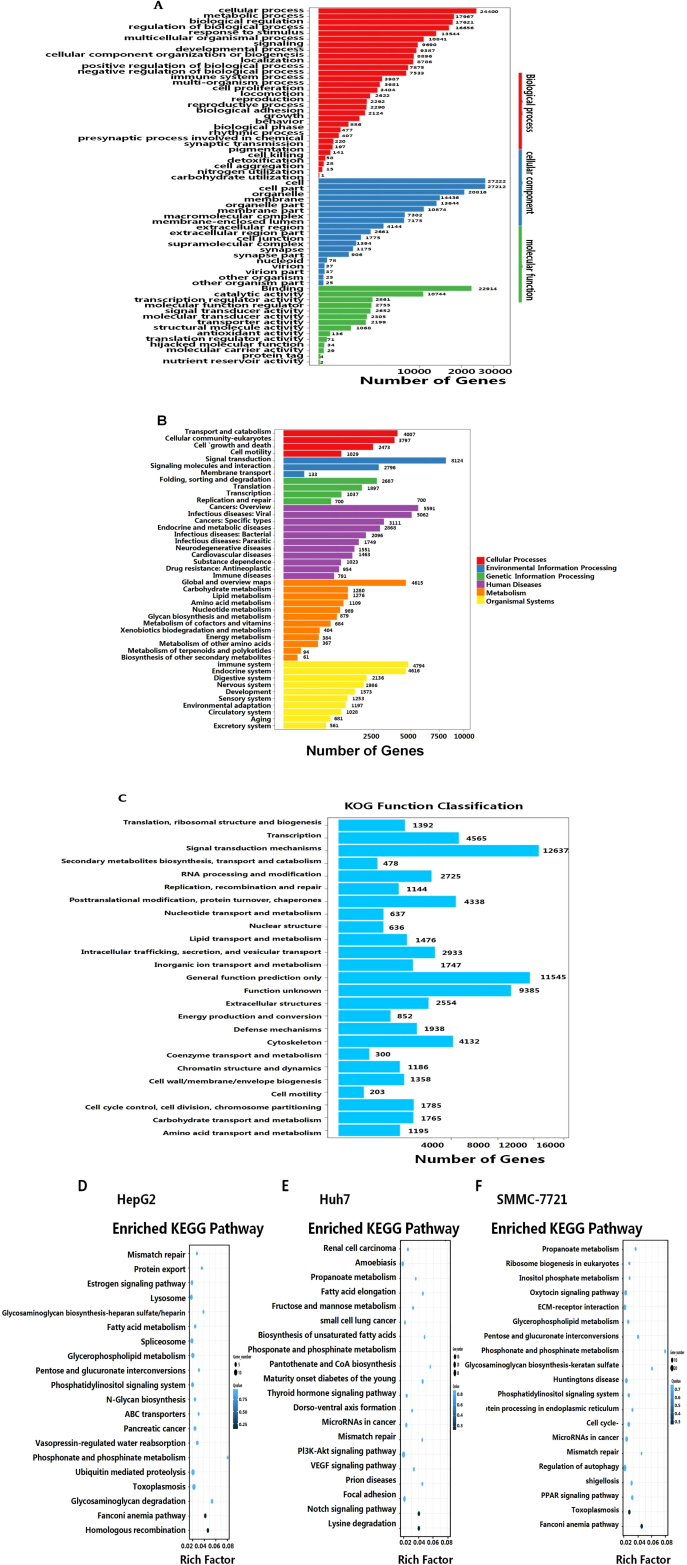
GO enrichment analysis of the target genes of the DE lncRNAs. **(A)** GO enrichments in the categories of biological process, cellular component, and molecular function. **(B)** LncRNA networks mainly associated with cellular processes, environmental information processing and genetic information processing, cancers, infectious diseases, and metabolism. **(C)** KOG functional classification. KEGG signaling pathway analyses of the differentially expressed lncRNAs-targeted mRNAs for: HepG2-FICZ vs. HepG2-blank **(D)**, Huh7-FICZ vs. Huh7-blank **(E)**, and SMMC7721-FICZ vs. SMMC7721-DMSO **(F)**.

Next, we analyzed the functional pathways of the differentially expressed RNAs following AHR activation in HCC cells. Gene Ontology (GO) analysis identified significant enrichment in 19 cellular component (CC) terms, 14 molecular function (MF) terms, and 30 biological process (BP) terms (**Figure 6A**). The analysis highlighted the prominence of genes involved in membrane components, developmental and immune processes, signal transduction, biological regulation, and metabolic pathways (**Figure 6B**).

The further classification of 72,686 transcripts using the EuKaryotic Orthologous Groups (KOG) database revealed 25 functional groups. The major categories included signaling mechanisms (12,637 transcripts) and general function prediction (11,545 transcripts), with additional representation in transcription (4,565 transcripts), post-translational modification, protein turnover, and molecular chaperones (4,338 transcripts), cytoskeleton (4,132 transcripts), and intracellular transport/secretion (2,933 transcripts). Less represented categories included cell motility (203 transcripts) and coenzyme transport/metabolism (300 transcripts) (**Figure 6C**).

In parallel, KEGG pathway analysis further elucidated the involvement of the AHR target genes in various cancer-related pathways. Specifically, these genes were prominently linked to PI3K-Akt, VEGF, Notch, and PPAR signaling pathways, as well as cancer-related microRNAs (**Figures 6D–F**). Additionally, AHR activation was associated with pathways related to fatty acid synthesis and metabolism, immune responses, and hormonal signaling, including estrogen, thyroid hormone, and oxytocin pathways. These results indicate that AHR activation significantly influences metabolic and signaling pathways pertinent to cancer progression, although the detailed mechanisms and downstream targets remain to be elucidated. This comprehensive classification underscores the broad impact of AHR activation on a diverse array of cellular functions and processes.

### Interaction analysis of lncRNA-mediated regulation in HCC

**Figure 7:**
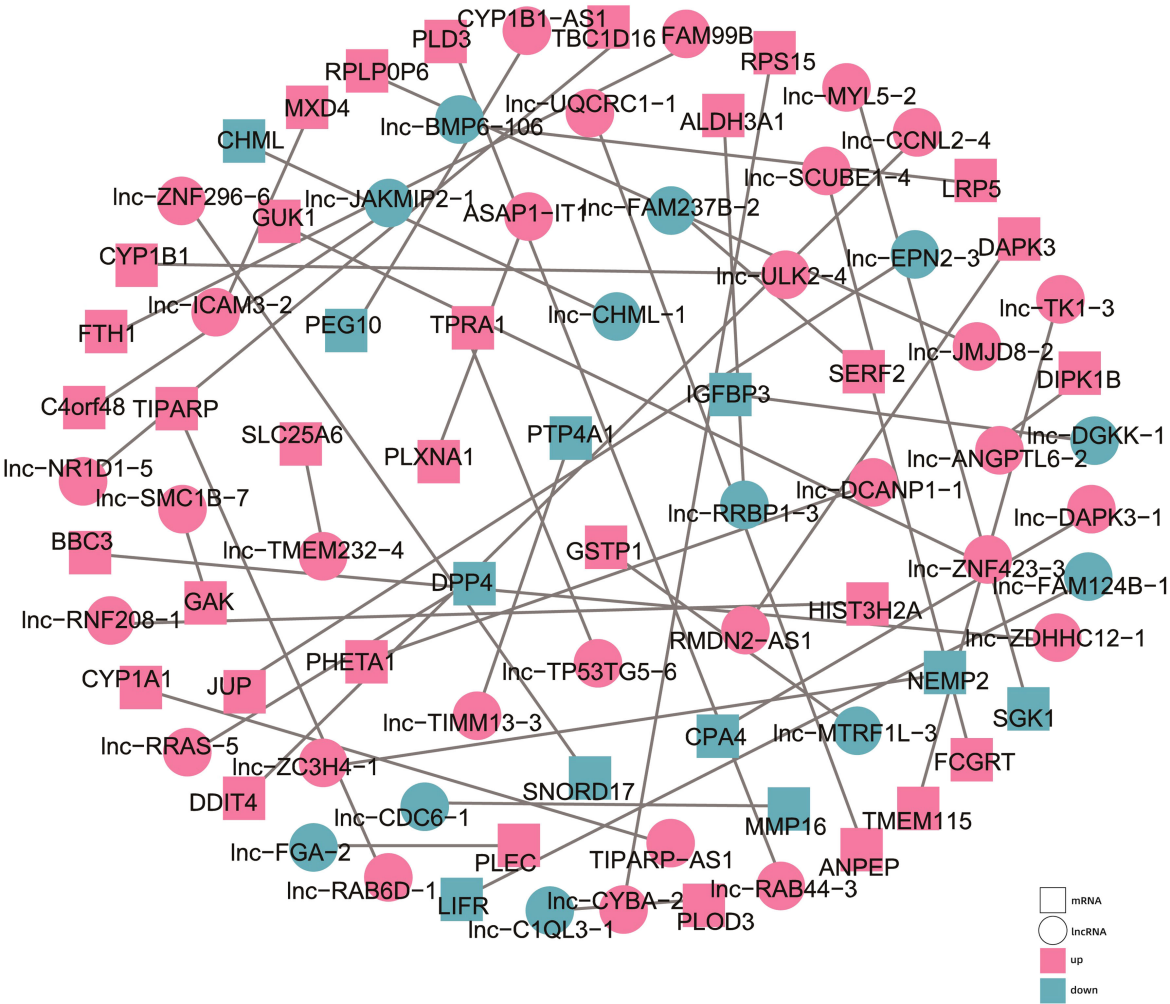
Diagram of the co-expression network between the differentially expressed lncRNAs and mRNAs. The circles represent the lncRNAs, while the squares represent the mRNAs. Purple represents upregulated, and blue represents downregulated.

To explore the regulatory roles of the candidate lncRNAs, we constructed a functional network centered around the differentially expressed (DE) lncRNAs and mRNAs. We screened the regulatory relationships between the DE lncRNAs and mRNAs in the TCGA and TargetScan databases and then visualized their co-expression network with Cytoscape (**Figure 7**).

We identified several significant co-expression pairs. For instance, *RMDN2-AS1* was found to be co-expressed with death-associated protein kinase 3 (*DAPK3*), *FAM99B* with ferritin heavy chain (*FTH1*), and *ASAP1-IT1* with plexin A1 (*PLXNA1*), a plasma membrane protein regulated by low-density lipoprotein receptor-related protein 1 (*LRP1*). Other notable associations included *TP53TG5-6* with transmembrane protein adipocyte-related 1 (*TPRA1*), *lnc-BMP6-106* with LDL receptor-related protein 5 (*LRP5*), and *DGKK-1* with insulin-like growth factor binding protein 3 (*IGFBP3*). Notably, these genes, and consequently their interacting lncRNAs, are involved in the regulation of lipid metabolism, and hence may potentially contribute to the lipid metabolic abnormalities observed in HCC cells. These findings aligned with our KEGG pathway analysis, which highlighted the impact of AHR activation on metabolic pathways, particularly those related to glucose and lipid metabolism.

Overall, our co-expression network analysis suggested that AHR activation modulates glucose and lipid metabolism in HCC at the transcriptional level. This network provides insights into the potential mechanisms underlying hepatocarcinogenesis and suggests directions for future functional studies aimed at understanding the role of lncRNAs in cancer metabolism.

### Prognostic relevance of the key AHR-related lncRNAs in HCC

To investigate the prognostic significance of the AHR-dysregulated lncRNAs in HCC, we conducted survival analysis using data from The Cancer Genome Atlas (TCGA). We analyzed the expression profiles of all the differentially expressed lncRNAs following AHR activation in 424 TCGA HCC samples and integrated the associated clinical data. Through this analysis, we identified 10 lncRNAs with significant survival implications and evaluated their potential impact on patient outcomes.

**Figure 8:**
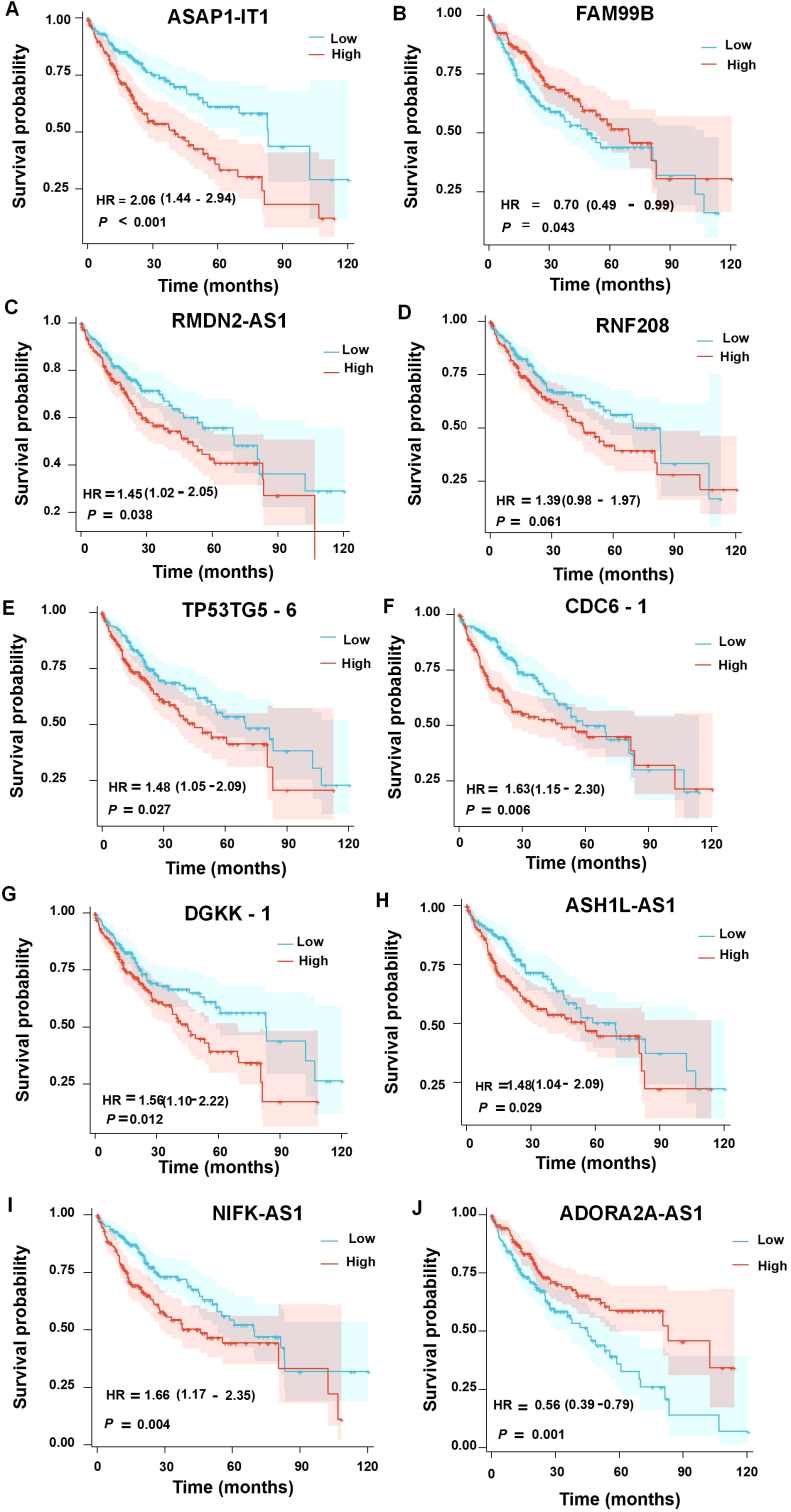
Survival analysis of the upregulated **(A–E)** or downregulated **(F–J)** differentially expressed lncRNAs in the prognosis of HCC patients.

Our analysis revealed that several of these lncRNAs were significantly associated with the prognosis in HCC patients (**Figure 8**). Specifically, higher expression levels of *ASAP1-IT1*, *RMDN2-AS1*, *RNF208*, and *TP53TG5-6* were correlated with a poorer overall survival, while elevated *FAM99B* expression was linked to improved outcomes. In contrast, among the downregulated lncRNAs, *CDC6-1, DGKK-1*, *NIFK-AS1*, and *ASH1L-AS1* were associated with an unfavorable prognosis, whereas a higher expression of *ADORA2A-AS1* was related to better patient survival.

Notably, *ASAP1-IT1*, *RMDN2-AS1*, and *TP53TG5-6* showed a particularly strong correlation with reduced overall survival in primary liver cancer patients, emphasizing their potential role as biomarkers for prognosis. These findings complement our earlier results, which implicated these lncRNAs in AHR-mediated regulatory networks, highlighting their dual importance in both disease progression and prognosis.

In summary, these AHR-dysregulated lncRNAs offer valuable insights into the molecular mechanisms of hepatocellular carcinoma and present potential targets for future therapeutic strategies.

## Discussion

In the present study, we investigated the role of AHR activation in HCC, particularly its regulatory effects on long non-coding RNAs (lncRNAs) and mRNAs, using the high-affinity ligand FICZ. Our results demonstrate that AHR activation leads to widespread changes in gene expression, particularly in pathways linked to glucose and lipid metabolism. These findings offer new insights into AHR’s involvement in metabolic reprogramming and its potential implications in HCC progression.

A central observation of our study was the significant dysregulation of metabolic pathways, which underscores AHR’s role in cellular metabolism. Metabolic reprogramming, particularly changes in lipid and glucose metabolism, is a hallmark of cancer progression, and is vital for supporting the energy demands of tumor cells (21, 22). Several of the differentially expressed lncRNAs and mRNAs identified in our analysis were associated with these metabolic processes. For instance, the co-expression of *lnc-ASAP1-IT1* with *PLXNA1*, a protein involved in membrane signaling, suggested the existence of potential regulatory interactions that contribute to metabolic shifts within the tumor microenvironment (23). Similarly, *lnc-DGKK-1* co-expression with *IGFBP3* highlighted the interplay between AHR activation and lipid metabolism, as *IGFBP3* has been implicated in promoting lipogenesis in hepatocytes (24). These metabolic alterations, including enhanced lipogenesis and glucose uptake, are crucial for tumor cell proliferation and survival, suggesting that AHR activation may facilitate tumor growth by promoting metabolic flexibility (25).

The lncRNA–mRNA co-expression network constructed in our study further illustrated the intricate regulatory landscape modulated by AHR activation. Key lncRNAs, such as lnc-*RMDN2-AS1*, co-expressed with *DAPK3*, suggest a role in apoptosis regulation—a critical pathway that is often disrupted in cancer (26). Another important co-expression pair comprised *FAM99B* and *FTH1*, pointing to a potential involvement in iron homeostasis and oxidative stress response, both of which are altered in cancer cells (27). These co-expression relationships not only deepen our understanding of how AHR modulates gene networks but also suggest that these lncRNAs could serve as regulatory nodes in tumorigenic processes, including metabolic adaptation, apoptosis evasion, and immune modulation.

Our findings also underscore the prognostic potential of several AHR-related lncRNAs. Survival analysis revealed that higher expression levels of *ASAP1-IT1*, *RMDN2-AS1*, and *TP53TG5-6* were associated with a poor prognosis in HCC patients, suggesting their utility as prognostic biomarkers. Conversely, a higher expression of *FAM99B* was correlated with better patient outcomes, illustrating the diverse roles that lncRNAs may play in tumor progression. These observations align with the growing evidence that lncRNAs can serve as key regulators of oncogenic pathways, influencing processes such as cell proliferation, migration, and immune evasion. The identification of AHR-related lncRNAs with prognostic significance reinforces the idea that they could be valuable targets for therapeutic intervention in HCC.

AHR is a ligand-activated transcription factor and environmental sensor (28). The AHR–CYP1–FICZ axis was demonstrated to be involved in CYP1A1 overexpression (29). It has also been reported that dietary flavonoids and tryptophan are metabolized into the potent AHR ligand FICZ, triggering AHR nuclear-cytoplasmic activation (30). Immunofluorescence and RNA-seq confirmed that AHR activation in liver cancer cells leads to nuclear translocation and gene regulation. GO clustering and KEGG pathway analyses revealed that FICZ-activated AHR promotes the expression of glucose and lipid metabolism-related genes. Although we did not directly study the AHR–lncRNA–metabolic axis, our findings suggest that modulating lncRNA expression through beneficial AHR ligands could provide a strategy to improve patient outcomes.

One limitation of this study to note is the selected treatment duration of 24 h, which, while effective for capturing early AHR activation and lncRNA responses, may not fully reflect the long-term AHR–lncRNA dynamics. Extending the treatment duration could provide additional insights into sustained or delayed regulatory effects and reveal further downstream interactions. Future studies could explore longer treatment periods to better understand the temporal dynamics of AHR-regulated lncRNAs. Moreover, the variations in AHR activation levels among the different HCC cell lines used in this study may reflect underlying differences in the HCC subtypes or stages. This variability could have influenced the observed AHR–lncRNA interactions and lipid metabolic effects, potentially acting as a confounding factor. Future studies should explore a broader range of cell lines and patient samples to account for this heterogeneity.

## Conclusions

Our study provides novel insights into the regulatory role of AHR in HCC, particularly its influence on lncRNAs and mRNAs involved in metabolic processes. The dysregulation of the glucose and lipid metabolism pathways highlights how AHR activation promotes tumor metabolic reprogramming, a crucial factor in tumor progression. Additionally, the identification of key AHR-related lncRNAs with prognostic significance suggests their potential as biomarkers and therapeutic targets in HCC. The interplay between AHR and lncRNAs may have important clinical implications in HCC treatment, particularly through its influence on tumor progression and metabolic reprogramming. Given that AHR regulates key oncogenic pathways and lncRNAs serve as crucial modulators of gene expression, targeting AHR–lncRNA interactions could provide novel therapeutic strategies. Such an approach may help modulate tumor metabolism and drug resistance, offering new avenues for HCC intervention.

## Data Availability

The sequencing data presented in the study are deposited in the national center for biotechnology information (NCBI), BioProject accession number PRJNA550009.
